# Uranium in Ohio, USA Surface Waters: Implications for a Fertilizer Source in Waters Draining Agricultural lands

**DOI:** 10.1038/s41598-020-61922-2

**Published:** 2020-03-20

**Authors:** W. Berry Lyons, Christopher B. Gardner, Susan A. Welch, Samantha Israel

**Affiliations:** 1 School of Earth Sciences, The Ohio State University, Columbus, OH 43210 USA; 2 Byrd Polar and Climate Research Center, The Ohio State University, Columbus, OH 43210 USA

**Keywords:** Hydrology, Geochemistry

## Abstract

Synthetic fertilizer is a potential source of uranium to natural waters, yet evidence is lacking. We analyzed dissolved uranium concentrations in lakes, reservoirs, and rivers in Ohio, USA during the summer of 2017. All water bodies drain areas of extensive agriculture where phosphate-rich fertilizer is applied. Uranium concentrations ranged from 0.3 to 3.9 µg L^−1^, with the lowest concentrations observed in the most offshore Lake Erie samples. These results, especially when placed in the context of previous work on both surface and groundwater, suggest that dissolved uranium concentrations in this water emanating from agricultural lands are higher than background, and uranium should be categorized similarly to nitrate and phosphate in that it originates in part from fertilizer application.

## Introduction

The impact of modern agricultural practices on water quality is well documented. The addition of N and P-rich fertilizer can increase the flux of these macronutrients into aquatic systems causing eutrophication and hypoxia^1^. The oxidation of ammonia rich fertilizer can produce acid, which ultimately increases chemical weathering in the soils^2,3^ and enhances the flux of Ca, Mg, and HCO_3_ into streams and rivers due to carbonate amendment^4,5^. Long term fertilizer and lime amendments increase P, Ca, Mg, and K to agricultural soils over decades^6^.

Another impact of the addition of P-rich fertilizer on agricultural lands is the introduction of various trace elements associated with the original phosphoritic materials that are subsequently transferred to final commercial products during processing. The total U concentrations in P-rich fertilizers derived from phosphorites are particularly high, ranging 10–60 µg g^−1^ ^7^. The U concentrations in the water soluble fraction of synthetic fertilizers vary greatly, from <0.05 μg g^−1^ to 7.68 μg g^−1^ with NPK fertilizers having the highest concentrations^7^. Long-term studies have documented U increases in agricultural soils due to the addition of P-rich fertilizers^8^, including increases of 4.4 to 13.7% over a 60 year period in Germany^9^. An estimated ~14,000 tons of U have been added to agricultural lands globally since 1951, corresponding to an average increase of ~1 kg U per hectare^9^. The fate of this anthropogenically introduced U is important – does the majority remain associated with soil, or is a fraction solubilized through time and introduced to aquatic systems? This is a significant question for two reasons: (1) the biochemical toxicity of U has been estimated to be six orders of magnitude higher than its radiological toxicity, and it has been implicated in potential chronic disease, such as kidney malfunction^10^; and (2) under oxic conditions, U(VI) is readily soluble and has the potential to be transported from soils to surface or groundwater.

Limestone dissolution has been identified a primary control on U concentrations in rivers, and black shales and other U-bearing lithologic units can be additional important sources^11^. In its U(VI) oxidation state, U forms strong complexes with carbonates and other oxygenated ligands to form highly soluble species over a wide pH range^12^. The agricultural processes of carbonate amendment and nitrate addition can work together to increase U mobility through direct increases in alkalinity, abiotic nitrate oxidation of U(IV) in minerals to U(VI), and a variety of biotic oxidation reactions that both increase alkalinity and oxidize U(IV)^13–15^. The concentration of U in surface waters in agricultural watersheds represents a mixture of natural (geogenic) sources resulting from water-rock interaction, anthropogenic agriculture-related factors that increase U solubility, and possibly direct contaminants from fertilizer impurities.

**Figure 1:**
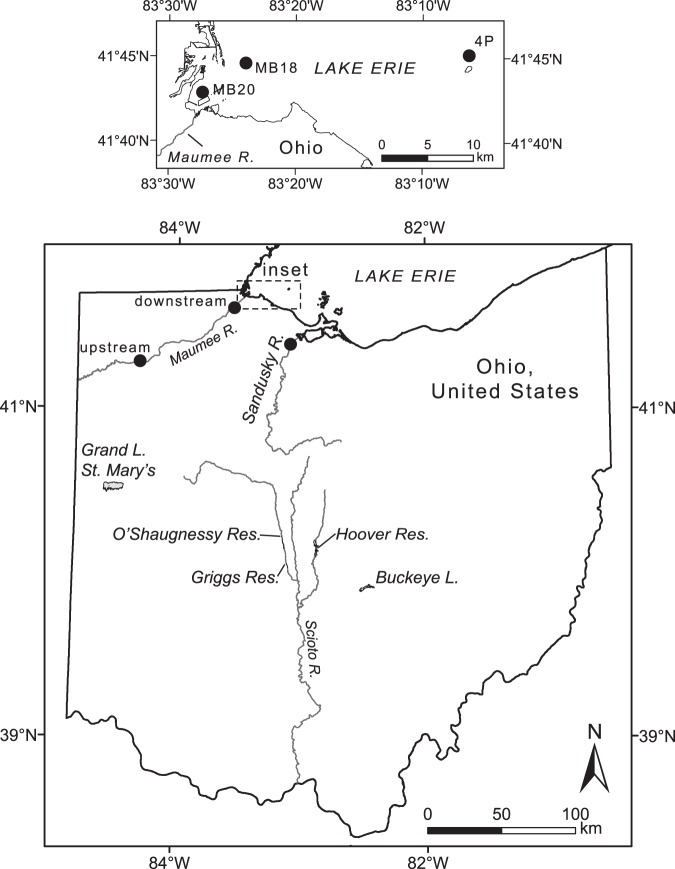
Water bodies and sampling locations in Ohio, United States.

We present dissolved U concentrations from surface waters in central and northwestern Ohio, USA, that drain agricultural landscapes during a late spring–summer temporal cycle. We include data from two rivers draining into Lake Erie, western Lake Erie waters, two reservoirs that have recently suffered from harmful algal blooms promoted by excess nutrient input, and three reservoirs that provide potable water to Columbus, Ohio, which also drain primarily agricultural lands (Fig. 1). All these waters, except those from Lake Erie, are in the Eastern Corn Belt Eco region and sit upon glaciated till plains with primarily row crop land use (i.e., corn and soya) agriculture.

## Materials and Methods

Water samples were collected by hand in summer, 2017 using ultraclean techniques for low-level sampling of trace elements. Samples for uranium analysis were filtered within 12 hours of collection using pre-cleaned plastic syringes and 0.45 μm Whatman polypropylene syringe filters pre-cleaned with 10% HNO_3_ and rinsed multiple times with DI water. Aliquots for major ions and major nutrients were collected similarly, but filtered using plastic filter towers through 0.4 μm Nuclepore filters. Major cations were analyzed using a Dionex DX-120 ion chromatograph (IC), and major anions were analyzed using a Dionex ICS-2100 IC^16^. Bicarbonate concentrations were calculated by the difference between total cation and anion concentrations in equivalents. Uranium measurements were made using a ThermoFinnigan Element 2 ICP-SF-MS, with an internal Indium standard (10 µg L^−1^). Measurement accuracy was checked against the TMDA 64.2 standard (Trace Elements in Water, Environment Canada) diluted 200:1 to match sample concentrations, and was found to be within 3.7% of the reference value. Replicate determinations of standards yielded a precision of ±2.7%. All field blanks and filtration blanks (our best DI water processed as samples) were below the limits of detection.

## Results and Discussion

**Figure 2:**
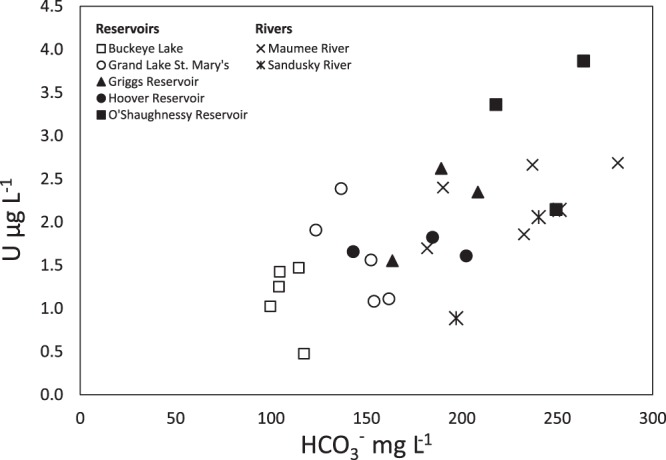
Uranium concentrations vs. calculated bicarbonate concentrations in reservoirs and rivers in Ohio.

Dissolved U concentrations range from 0.3 to 3.9 µg L^−1^, with the lowest values measured in offshore samples from Lake Erie, and the highest values in O’Shaughnessy and Griggs Reservoirs and the Maumee River (Table 1). U concentrations at the offshore (4 P) Lake Erie location remained relatively constant at 0.4 ± 0.07 µg L^−1^ while U in the southern reservoirs, two rivers and the O’Shaughnessy and Griggs Reservoirs varied considerably from the late spring through early summer (Table 1). Except for the offshore Lake Erie samples, all the samples were higher than the global mean river U concentration of 0.19 µg L^−1^ ^11^. Uranium concentrations exhibited a positive relationship with bicarbonate concentrations (r^2^ = 0.44, *p* = 2 × 10^−4^) across the reservoir and stream data (Fig. 2), indicating that the U is likely complexed with carbonate species. However, this relationship is not present at the level of individual sites, suggesting that U in these surface water is not derived solely from carbonate lithologies.

**Table 1:** U concentrations in Ohio water bodies collected in summer 2017 (µg L^−1^).

	23-May	5-Jun	19-Jun	21-Jun	6-Jul	10-Jul	24-Jul	26-Jul	31-Jul	15-Aug	21-Aug	23-Aug	6-Sep	Mean
***Reservoirs***														
Buckeye Lake	1.5	1.4				0.5	1.0			1.3				**1.1**
Grand Lake St. Mary’s	2.4	1.9				1.1	1.1			1.6				**1.6**
Griggs Reservoir	2.3	1.6								2.6				**2.2**
Hoover Reservoir	1.6	1.7								1.8				**1.7**
O’Shaugnessy Reservoir	3.9	2.1								3.4				**3.1**
***Rivers***														
Maumee River downstream			2.7									1.9		**2.3**
Maumee River upstream			2.7									2.4	1.7	**2.3**
Sandusky River			0.9									2.1	2.1	**1.7**
***Lake Erie***														
MB20 (Maumee Mouth)								4.4	1.2					**2.8**
MB18 (Maumee Bay)				0.5										**0.5**
4P (offshore)				0.3	0.3			0.4	0.4		0.4			**0.4**

Although the subsurface lithologies of these watersheds consist of a wide range of Paleozoic sedimentary rocks, including carbonates and shales, they are mantled with an extensive drape of glacial tills. Much of the agricultural lands contain drainage tiles that can rapidly re-route surface runoff into local streams prior to recharging the deeper subsurface. Tile drains have been demonstrated to increase nutrient discharge to surface waters in Ohio^17^. Low groundwater recharge rates of 10–15 cm yr^−1^ are observed in the study area due to the presence of glacial tills^18^.

**Figure 3:**
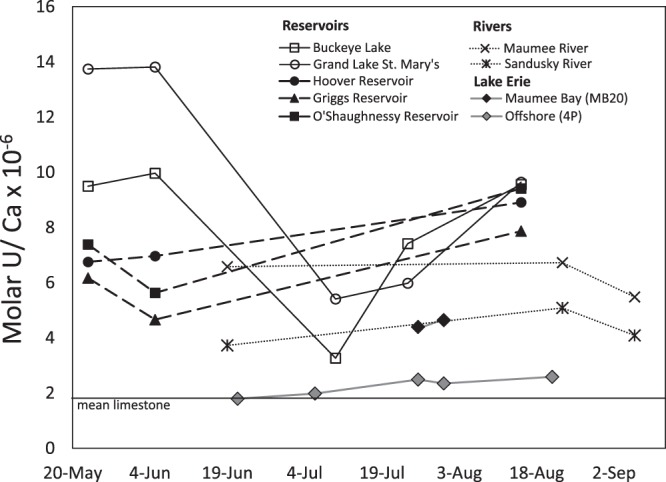
Molar U/Ca ratios in Ohio surface waters during summer, 2017. ‘Mean limestone’ is the mean U/Ca molar ratio in limestone^20^. Error bars associated with analytical uncertainty are approximately the size of the symbols.

Watershed lithology can exert an influence on riverine U concentrations as waters draining the carbonate and shale bedrock can have higher U values^19^. The watersheds of all but Buckeye Lake and the Hoover Reservoir do have underlying Paleozoic carbonates which could be sources of dissolved U. The molar U:Ca of our water samples and the average molar U:Ca ratio (1.8 × 10^−6^) in limestone^20^ are plotted in Fig. 3. Stream and reservoir samples and Lake Erie samples from Maumee Bay plot above the mean limestone value while offshore Lake Erie samples plot on or near the average, indicating that U is either preferentially solubilized from the carbonates, the Ohio carbonates have higher U:Ca than other Paleozoic limestones, or there is another source of U to these systems. Previous work suggests that as carbonate rocks are weathered, some of the U present is retained in the soil profile, primarily adsorbed onto ferric oxyhydroxides^21,22^. Others have argued that U:Ca ratios in waters draining carbonate rocks are constrained by the initial U:Ca in the rocks^23^, thus the preferential solubilization of U over Ca in these Ohio carbonates seems unlikely. Another major natural source of U in these soils is from the Devonian Ohio Shale found in glacial drift throughout central and west central Ohio^24^. Soils in Ohio are elevated in U compared to average U.S. soils, with values in central and parts of eastern Ohio of 2.7–6.0 µg g^−1^ ^24^, while the 50^th^ percentile concentration of soils from the continuous US is 2.0 µg g^−1^ ^25^. Although we cannot rule out the premise that these higher than mean global U fluvial concentrations are due, at least in part, to the dissolution of U-rich minerals in these Paleozoic rocks, we hypothesize that a significant portion of the dissolved U observed in these waters in Ohio is due to input from P-rich fertilizers.

We are not the first to suggest the application of P-rich fertilizer on agricultural lands increases the dissolved U in surrounding surface waters. Rivers draining cotton agricultural lands in Texas were found to have higher U concentrations than forested watersheds^26^. Others have found soil waters in agricultural lands and agricultural drainage waters have high dissolved U concentrations^27,28^, with concentrations as high as 2.4 µg L^−1^ in agricultural drainage waters in the Florida Everglades (compared to background values of <0.1 µg L^−1^)^29^. Elevated dissolved U concentrations have been noted in agricultural watersheds in Germany^30^ and Brazil^31^.

High concentrations of dissolved U have been observed in groundwaters in the intensely agricultural lands of the High Plains and the Central Valley, California, USA, where 10% of wells exceeded the US Environmental Protection Agency maximum contaminant level (MCL) of 30 µg L^−1^, with concentrations as high as 2674 and 5400 µg L^−1^ ^32^. These authors found that nitrate concentrations near the MCL were correlated to U, especially in shallower wells, and surmised that the U was produced from aquifer materials through nitrate-mediated U solubilization. Another possible interpretation of these data is that the dissolved U actually originated from the same fertilizer source as the nitrate, thereby being a direct, rather than an indirect, product of fertilizer application.

Shiller (1997) measured dissolved U concentrations in the Mississippi River in the range of 0.29 to 2.5 µg L^−1^ with means in two different years of 1.1 and 1.6 µg L^−1^ ^33^. The highest values are associated with the record 1993 flood on the lower Mississippi River. Shiller noted that unlike all the other metals measured, “hydrologic factors” were important for U, reflecting the importance of discharge in controlling its concentration.

The planting of row crops, especially soya, has greatly affected the hydrology of many parts of the U.S. Increased streamflow in Iowa has been attributed to the reduction of evapotranspiration in row crop agriculture and a subsequent increase in the shallow groundwater entering into streams^34^. Fifty percent of the increase in bicarbonate in the Mississippi river was attributed to this increase in agriculturally induced discharge, and any soluble element associated with agricultural practices is probably also affected by this process^35^. Tile drainage in Illinois increased nitrate fluxes 2.5 to 4 times above the non-tiled agricultural watersheds^36^. Enhanced artificial drainage could help fertilizer-added U bypass soil adhesion and discharge directly into surface waters.

We posit that enhanced fertilizer usage along with increased discharge through the vadose zone of agricultural lands has increased the U concentration, both in the shallow groundwater and surface runoff. In addition, there is a large amount of legacy fertilizer products still on the landscape, both in the soil and groundwater, that can serve as a long term continued source to surface waters in the future^37^. It should be strongly emphasized that the U concentrations observed in Ohio are far below the drinking water standard of 30 µg L^−1^. However, we do suggest that, like nitrate and phosphate, dissolved U should be monitored on a more regular basis in agricultural watersheds than it is currently.
